# Dynamics of the Central Bottleneck: Dual-Task and Task Uncertainty

**DOI:** 10.1371/journal.pbio.0040220

**Published:** 2006-06-27

**Authors:** Mariano Sigman, Stanislas Dehaene

**Affiliations:** **1**Unité INSERM 562, Cognitive Neuroimaging, Service Hospitalier Frédéric Joliot, CEA/DRM/DSV, Orsay, France; **2**Collège de France, Paris, France; Bremen UniversityGermany

## Abstract

Why is the human brain fundamentally limited when attempting to execute two tasks at the same time or in close succession? Two classical paradigms, *psychological refractory period* (PRP) and *task switching,* have independently approached this issue, making significant advances in our understanding of the architecture of cognition. Yet, there is an apparent contradiction between the conclusions derived from these two paradigms. The PRP paradigm, on the one hand, suggests that the simultaneous execution of two tasks is limited solely by a passive structural bottleneck in which the tasks are executed on a first-come, first-served basis. The task-switching paradigm, on the other hand, argues that switching back and forth between task configurations must be actively controlled by a central executive system (the system controlling voluntary, planned, and flexible action). Here we have explicitly designed an experiment mixing the essential ingredients of both paradigms: task uncertainty and task simultaneity. In addition to a central bottleneck, we obtain evidence for active processes of task setting (planning of the appropriate sequence of actions) and task disengaging (suppression of the plan set for the first task in order to proceed with the next one). Our results clarify the chronometric relations between these central components of dual-task processing, and in particular whether they operate serially or in parallel. On this basis, we propose a hierarchical model of cognitive architecture that provides a synthesis of task-switching and PRP paradigms.

## Abstract 2

The authors investigate the cognitive processes that constrain our ability to perform multiple mental tasks by clarifying the relationship between task chronometry and the dynamics of task selection.

## Introduction

Several cognitive theories share the hypothesis that while most mental and neural operations are modular, certain controlled processes require a distinct capacity-limited “central executive” or “global workspace” system that can establish flexible links amongst existing processors [ 1– 5]. The dynamics of engagement and disengagement of central processing is manifested in two different time scales. First, at a time scale of a single trial or event (few hundred milliseconds), dual-task interference is observed when processing simultaneously or quasi-simultaneously two different streams. This happens, among other examples, when two masked stimuli are presented at a short stimulus onset asynchrony (SOA) [ 6] (the attentional blink) or when participants are asked to process two consecutive tasks at a short time interval (psychological refractory period or, in short, PRP) [ 7– 12]. A dynamic trace of central limitation is also manifested at a slower time-scale (seconds to minutes) in the inability to rapidly switch the control processes that harness together independent processing modules [ 13]. This effect is most evident in task-switching paradigms, which show, using a variety of different experimental manipulations, that reaction times increase when participants change between different task configurations [ 14]. A theoretical discussion has taken place on understanding whether task switching costs result from the insertion of a stage-like executive control process that operates only on switch trials [ 15– 17], or rather, from a carryover effect (inertia) of the previous task that establishes a conflict between previous and current task settings [ 14, 18, 19]. Even though a theoretical model has shown that task carryover can account for most task-switching effects [ 20], it is very likely that the operation of setting for a novel task should be observable, and both preparation of the new task set and disengagement have been shown to have a role in task-switching costs [ 21] . Similarly, an executive process of engagement and disengagement has been observed in the cognitive act of shifting attention from one place in the visual field to another [ 22, 23].

Although the dual-task and the task-switching literature has addressed independently the dynamics of central coordination between processing modules, there is an apparent contradiction between them. A classic result of dual-task interference is that when two tasks are presented simultaneously or at a short SOA, there is a delay in the execution of the second task, but performance of the first task is unaffected [ 9, 11, 24]. This implies that the central processing stages are dedicated sequentially to each task as opposed to spread uniformly between both concurrent tasks (which would yield a delay in both tasks). As SOA increases, the response time to the second task decreases with a slope of −1 until a non-interference regime is reached at an SOA of about 500 ms (dependent on the response time to task 1), after which performance on the second task becomes independent of SOA [ 9, 25]. These findings have been consistently explained in terms of a sequential model with three processing stages: (1) An initial perceptual stage that can be carried out in parallel and does not contribute significantly to the variance, (2) a central decisional stage that establishes a processing bottleneck and reflects a stochastic integration of evidence, thus providing a major contribution to trial-to-trial variability, and (3) a motor stage of response execution that is parallel and invariant. Here we refer to this model as the *passive bottleneck model,* because it is based on two fundamental principles: Access to the central space is determined on a first-come, first-served basis, and there is an immediate switching between the central processes of both tasks. These principles are in apparent contradiction with the task-switching literature, since they suggest that the sequencing of central operations, disengaging from one stimulus-response configuration and engaging in the other one, is done passively and at no cost.

Recently, an observation was made that suggests a possible reconciliation of the passive bottleneck model with the well-known task-switching cost: Responses to the first task in the PRP paradigm, although independent of SOA, are slower than when performing the task in isolation [ 26, 27]. We propose that this may be related to a control stage engaged before performing the first task in order to prepare for the instruction of performing the two tasks in a specific order. This additional processing time needs to be incorporated into a model of task architecture in order to predict quantitatively the response time distributions in experiments in which the order of the presentation of the two tasks, their relative offset, and their complexity are changed [ 27]. In addition, it has been shown that task switching may affect central interference. For example, in a cross-modal attentional blink experiment, there is no blink unless a task switch accompanies the modality switch [ 28]. Yet, it is not understood whether these manifestations of central processing involve shared or distinct mechanisms, or how to incorporate them in a model of cognitive architecture.

Here we investigate the precise relationship between task switching and the processing bottlenecks within each task. As in all PRP experiments, we engage participants in a double-task experiment in which they have to respond rapidly to a number comparison and a tone discrimination task. The main novelty is that both the task order and the SOA between the two stimuli are unpredictable, implying that participants cannot prepare beforehand for a specific task. Our experiment has two main objectives: (1) Understanding the dynamics of task selection: Is task response order decided upon stimulus presentation, or rather after perceptual processing has been completed, at the time the processing stream accesses the central bottleneck? and (2) can we generate a unified model that will incorporate in its architecture the passive bottleneck model of processing and the task-switching literature? Or, in other words, what is the dynamics of task choice and how does this interact with the different stages of processing of each task?

## Results

Participants were asked to perform a dual task. One of the two tasks was presented visually and involved a number comparison: Participants indicated with the right hand whether a two-digit number presented on the screen was larger or smaller than 45. The other was a tone discrimination task which involved deciding whether the frequency of a 150-ms pure tone was high (880 Hz) or low (440 Hz). The order of presentation of both stimuli changed randomly from trial to trial, and the SOA was sampled in 15 different values from −1,000 to 1,000 ms. (SOA is defined as the difference between the presentation of the tone and of the digit, and thus negative values correspond to trials in which the digit was presented before the tone.) In addition, in 11.76% of the trials (four out of 34), only one of the two stimuli was presented to allow collecting a single-task baseline performance. Participants were instructed to respond as fast as possible to each presented stimuli and could freely decide to which stimulus to respond first. The number task was our main task of study, and was manipulated using two independent factors: *notation* (whether the number was presented in Arabic digits or in spelled words) and *distance* (the numerical distance between the presented number and 45). The tone task was never varied throughout the experiment.

Throughout the manuscript we will refer to trials as *“number presented first”* or *“tone presented first,”* depending on the presentation order of the stimuli and as *“tone responded to first”* and *“number responded to first,”* depending on the order of the response chosen by participants (irrespective of the order of presentation). Note that although correlated, the order of presentation and of response is not necessarily identical for each trial and thus, we will refer without redundancy to *“number presented and responded to first”* (and *“tone presented and responded to first”*).

### Task Selection: Gating Access to Central Processors

A first basic observation in our task is that instructions notwithstanding, participants appear unable to perform the two tasks simultaneously. Examining the distribution of the response time to the second task (RT2) minus the response time to the first task (RT1), designated RT2-RT1 (the difference in response times [RTs] to the two tasks), we observe essentially no concomitant responses by the two hands: Even when the stimuli occur simultaneously (SOA = 0), only a very small percentage of trials (0.8%) exhibit an absolute value of RT2-RT1 below 150 ms. This implies that performance of the two tasks is sequential.

We can then ask what determines the participants' choice of which task to perform first (as indicated by which of the two responses occurs first). The dependence of task choice on SOA ( Figure 1A), which follows a sigmoidal relation, indicates that selecting which task to respond first is determined, within a certain temporal jitter, by presentation order, providing a first line of evidence for a bottom-up contribution to task choice. However, there is also a bias for responding first to the number task (visual modality, right hand). At SOA = 0 (simultaneous presentation), “tone responded first trials” corresponded to only 33.7% of the total. The SOA value giving an unbalanced choice (50% responses to each task), calculated by linear extrapolation of the distribution and henceforth briefly referred to as 50%SOA, is 103 ms (see Figure 1). The temporal interval from an SOA of 80% of “number responded to first trials” to a SOA of 80% of “tone responded to first trials” is 373 ± 187 ms. Thus, from these first results, we conclude that task selection is guided by a bottom-up process with both noise and bias.

**Figure 1:**
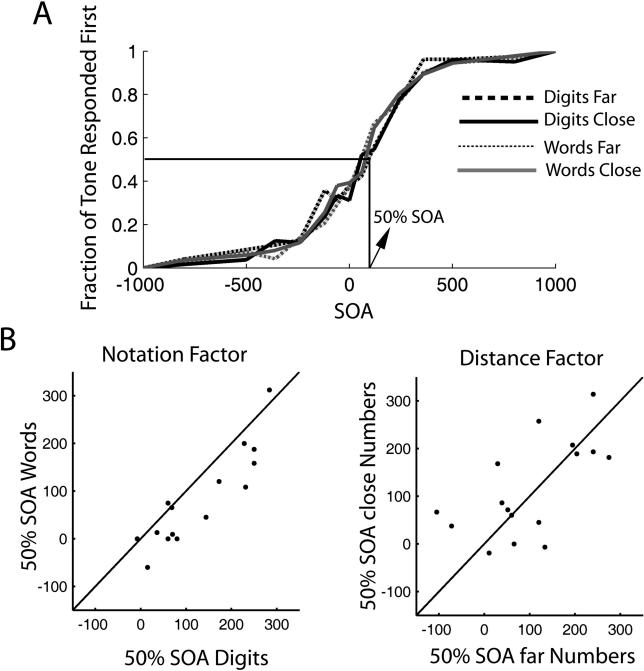
Determinants of Task Choice (A) Average proportion of trials in which participants responded first to the tone task as a function of SOA. The center of the distribution (50% of responses to each modality) is at 103 ms, indicating that there is a bias to respond first to the number task. The notation manipulation results in a significant change of the 50%SOA (37.8 ms), but the distance manipulation does not. (B) Participant by participant measures of the 50%SOA computed separately as a function of notation and distance. The line corresponds to the identity line. For the notation factor, almost all points lie on one side of the curve, indicating that 50%SOA is systematically larger for Arabic digits. For the distance factor, all values are scattered on both sides of the identity line.

Next we tested whether the decision of which task to respond to first is triggered by stimulus onset or, rather, after perceptual processing has been completed. These two alternatives can be distinguished by studying the impact of the experimental manipulations in the number task on the distribution of task selection. The passive bottleneck model predicts that whichever stimulus first reaches the central bottleneck is processed first, and thus that slowing perceptual processing would affect task choice. To test this, we capitalized on the fact that response times to a number comparison task are slower when numbers are presented in spelled-out words than in Arabic digits and, in a comparable amount, when the numeric distance to the target is shorter [ 29, 30]. To verify this finding in this particular experimental setting, we performed an analysis of variance (ANOVA) for RTs from the single-task condition, with participants as a random factor, and distance and notation as within-participant factors. For the distance manipulation, the mean difference between *far* and *close* numbers is 112 ms ( *F*(1,16) = 9.51, *p* = 0.008), for the notation manipulation the mean difference between spelled words an Arabic digits is 86 ms ( *F*(1,16) = 15.62, *p* = 0.001) and the interaction between these two factors is not significant ( *F*(1,16) = 0,73, *p* = 0.4), revealing an additive effect. In addition, we have previously shown that the notation manipulation affects a parallel perceptual stage whereas the distance manipulation affects a serial central stage [ 27].

If task order is decided at the onset of stimulus presentation, then manipulating the number task should not affect the distribution of first-task selection. On the contrary, if deciding which task to respond to first is undertaken once peripheral perceptual processing has been completed, then the notation manipulation should affect the value of 50%SOA, whereas the distance manipulation should have no effect. To decide between these two possible alternatives, we measured the 50%SOA as a function of the different manipulations. The notation manipulation resulted in a significant shift of the 50%SOA (mean difference between 50%SOA of Arabic digits and words: 37.8 ms [ *t* = 3.5, *df* = 16, *p*-value = 0.003, 95% confidence interval (CI): 14 to 60 ms]) Note that this shift is in the expected direction since the 50%SOA for Arabic digits is more positive than for spelled-out words, indicating that when perceptual treatment of the visual object is performed faster, the tone has to be presented earlier to reach an equal-choice situation. On the contrary, the distance manipulation (while having a bigger impact on the number task RTs) did not result in a significant change of the 50%SOA: (mean difference between RTs of far and close numbers: −9 ms ( *t* = −0.44, *df* = 16, *p-*value = 0.66, 95% CI: −51 to 33 ms)

### Response Time to the First Task

In a classic PRP experiment, two tasks have to be performed in succession as rapidly as possible. The order of the two tasks is held constant, and the SOA is the main experimental parameter. A classic result is that the response time to the first task is independent of SOA, even though its absolute value may be augmented relative to the time it takes to perform the first task in isolation [ 9– 11, 27]. Here, we have departed from this classic paradigm in that the task order is not held constant, and thus participants have to decide which task to perform first. Contrary to classic PRP experiments, in this situation of unpredictable task order, we observe an important difference in response time between long and short SOAs for the task responded to first. To quantify this, mean RTs were calculated for each SOA and each participant, and then submitted to ANOVAs with participants as a random factor and delay as a within-participant factor. For the *number presented and responded to first* and considering all SOAs from 0 to 1,000 ms ( Figure 2A, left side of the panel), there was a significant effect of SOA ( *F*(6,16) = 6.63, *p* < 5 × 10 ^−7^). Interestingly, as can be seen in Figure 2A, this effect showed a sharp transition close to 300 ms. Indeed, when the ANOVA was restricted to SOA > 300 ms for the *number presented and responded to first*, the effect of SOA was not significant ( *F*(3,16) = 0.91, *p* > 0.4). The mean values of the response times to the number task when presented and responded to first are 1,048 ms for SOA < 300 ms and 1,125 ms for SOA > 300 ms. A *t-*test comparing these two groups (SOA < 300 and SOA > 300) is highly significant ( *t* = −4.80, *p* < 1.6 × 10 ^−6^, CI: 43 to 103 ms). For the *tone presented and responded first* ( Figure 2A, right side of the panel) when considering all SOAs from 0 to 1,000 ms, the effect of SOA is highly significant ( *F*(6,16) = 25.93, *p* < 5 × 10 ^−16^). The effect of delay is still significant when restricting the analysis to SOA > 300, ( *F*(3,16) = 7.15, *p* < 10 ^−4^). The mean value of the response time to the tone task when presented and responded to first is: 1,142 ms for SOA < 300 ms and 964 ms for SOA > 300 ms. A *t*-test comparing these two groups (SOA < 300 and SOA > 300) is highly significant ( *t* = 10.56, *p* < 2.2 × 10 ^−16^, CI: 144 to 210 ms). The mean response times for the single tasks are 984 ms for the tone task and 1,011 ms for the number task. These values are significantly larger than the response times when both tasks are presented second at a SOA of 1,000 ms (855 ms for the tone task and 924 ms for the number task), indicating that even when the task is presented in isolation, response times are slower when participants have less certainty of the task they will have to perform and the time at which it will be presented.

**Figure 2:**
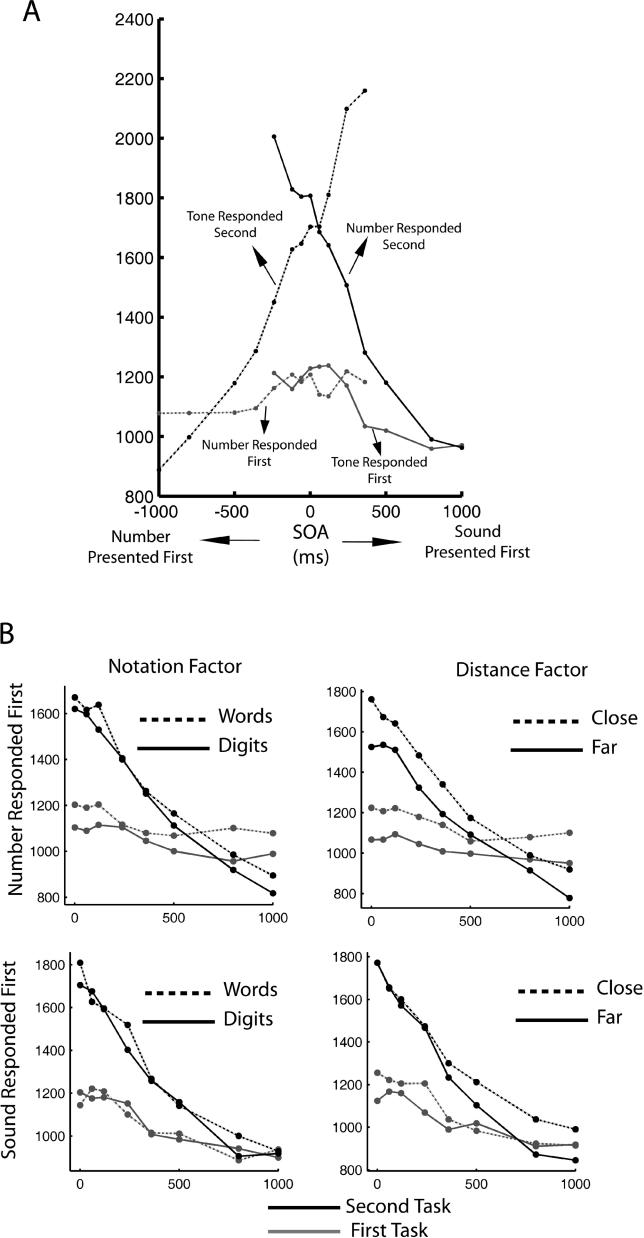
Mean Response Times: A Trace of Different Sources of Interference (A) Response times to the first and second tasks as a function of SOA. The labels indicate whether the first task corresponds to the number task (mostly for negative SOAs) or to the tone task (mostly for positive SOAs). Two main effects are observed: (1) Responses to the first task are slowed for small SOAs (<400 ms). This increase shows a fairly sharp transition. (2) Response times to the second task decrease linearly for small SOAs, reflecting a processing bottleneck. (B) When the number task is presented and responded to first (top two panels), the manipulations of the number task have an additive effect (independent of SOA). This effect propagates to the tone task. When the tone task is presented and responded to first (bottom two panels), it is not affected by manipulations of the number task (as predicted by a sequential processing model)

We next addressed the effects of all experimental manipulations on the first task ( Figure 2B) by performing ANOVAs with participants as a random factor, and delay and either distance or notation as a within-participant factor. A triple ANOVA with distance, notation, and SOA as within-participant factors could not be performed since there were not enough trials per condition for small SOAs. The detailed results of those ANOVAs are reported in Table 1. In the text, we merely draw attention to the main points: First, as described previously, regardless of the order of response, we observe a significant effect of SOA. Second, we observe an effect of both manipulations on the number task on “number presented and responded to first,” which does not interact with SOA (as expected in classic PRP experiments). Third, there is no effect of the manipulations of the number task on the tone task when it is presented and responded to first. An effect of SOA on RT1, additive on the manipulations of the first task, constitutes a first departure from the passive bottleneck model.

**Table 1:**
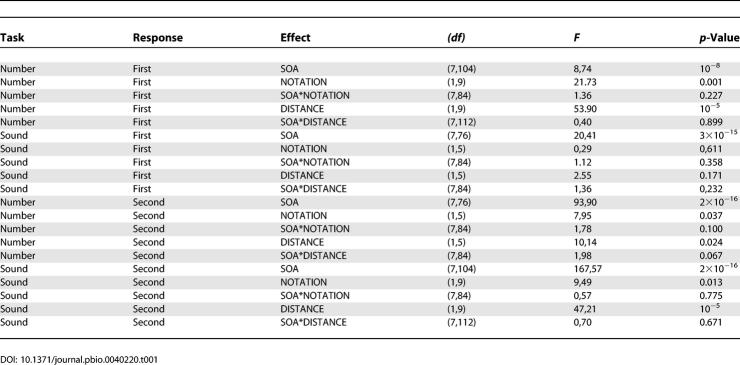
Results of ANOVAs

### Response Time to the Second Task

A classic result in PRP experiments is that RT2 decreases linearly with SOA, with a slope of −1, for small values of SOA (<400 ms). This effect has been explained in terms of a processing bottleneck. The central process of the second task cannot be executed until central processes of the task 1 is completed and thus, the response to the second task is not locked to the onset of its corresponding stimuli, but rather to the response to the first task. Hence RT increases proportionally as SOA is decreased (the sooner it is presented the more it has to wait for its execution). Since here we found that the response time to the first task increases for small SOAs (see section before), we wanted to address whether this would constitute an additive effect. In this case, we would expect RT2 to decrease with SOA with a slope more negative than −1. Indeed, for small SOAs, ( *abs*(SOA) < 400) we find a linear decrease with SOA, with a slope significantly more negative than −1 for number responded to second (β = −1.3, *R ^2^* = 0.98; *t-*test for a difference with a slope of −1, *t* = 2.29, *df* = 16, *p-*value = 0.035). For tone responded to second, we find a slope more negative than −1, but not so significantly (β = −1.13, *R ^2^* = 0.96; *t* = 1.7399, *df* = 16, *p-*value = 0.101).

To understand the effects of the different experimental manipulations of the number task on the second task ( Figure 2B), we submitted RTs to an ANOVA with participants as a random factor, and delay and either notation or distance as a within-participant factor. We did this separately for the case *number presented and responded to first* (to study the propagation of effects to the tone task) and *number presented and responded to second* (to study the interaction between the effects of the manipulation and the interference with the tone task). In both cases, a triple ANOVA could not be performed since for small values of SOA, we did not have sufficient trials per condition.

When the tone task is presented and responded to second, we observe a significant effect of both manipulations. This effect does not interact with SOA, indicating that even for long SOAs (up to 1000 ms) manipulations of the number task propagate to the second tone task. In previous experiments, in which the task order was fixed, we observed a propagation of notation and distance manipulations on the number task, to a subsequent tone task. This effect vanished at an SOA close to 600 ms, when the second task was performed independently of the duration of the first task. Here, we observe a more extended range of interference, which is also reflected in the fact that even for long SOAs response times to the second task show a monotonic decrease. This extended range of interference is however expected, given the slow responses to the first task due to task ambiguity as reported previously.

When the number task is presented and responded to second, we observe a significant effect of both manipulations on RT2. Both effects, however, are not significant for small SOAs < 400 ms. This is reflected in a marginally significant interaction of either manipulation (more prominently the distance manipulation) with SOA. This result is different than what we had found when both tasks at presented in a fixed order [ 27]. In this case, the effect of notation was absorbed for short SOAs, which indicated that it affected a perceptual component, but the distance effect was present at all SOAs which corresponds to the manipulation of a central stage. The absorption of the distance effect for short SOA when the number task follows the tone task constitutes a second departure from the passive bottleneck model.

### Variability of the Response Times to Each Task and Correlations between Them

Up to this point, we have concentrated our analysis on mean response times. Yet, a characterization of the sources contributing to trial-to-trial variability is important to understand mental chronometry. Thus, here we study the impact of the different experimental manipulations on the dispersion of the response times. A study of the histograms of response times ( Figure 3) indicates that the dependence of response times to the first and second task on SOA results from different factors. For the second task, as SOA decreases, the distributions shift to the right and become wider. This is consistent with the passive bottleneck model, according to which this delay results from the insertion of a variable process (the bottleneck stage of task 1). For the task responded to first, the distributions show a wider distribution, but a similar starting point. This, in turn, is suggestive of a decision process which is always present but variable and becomes increasingly difficult when the two tasks are presented closer in time.

**Figure 3:**
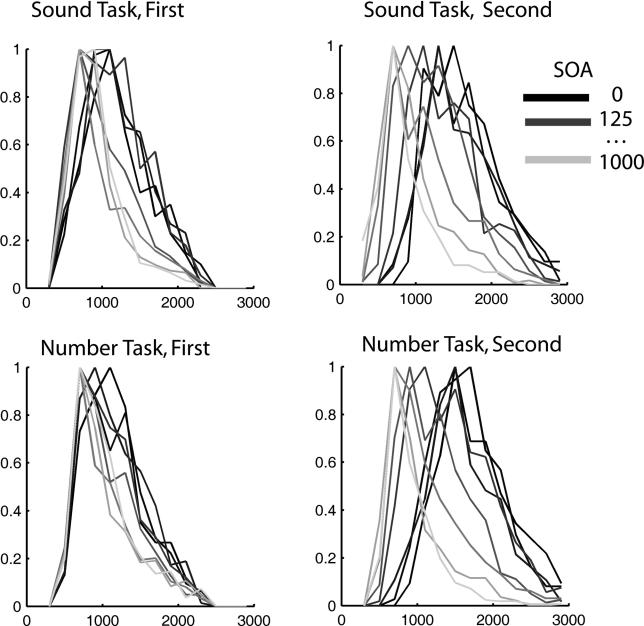
Distribution of Response Times for the First and Second Task: Delayed Decision or Inserted Stage? Distribution of response times to the first task (left) and second task (right). For clarity, all distributions were normalized to a peak of 1. In both cases, the mean response times increase with decreasing SOAs. From the distributions, it is seen that these effects result from qualitatively different changes. Although increases in the second task result from a delayed onset and widening of the distribution (consistent with the insertion of a variable delay in every single trial due to the processing bottleneck), the onset of the distributions of the first task, for different SOAs, is unchanged. The latter indicates that the increase in the mean response times with SOA for RT1 does not result from the inclusion of a processing stage for each trial. Rather, it results either from a variable lengthening of an already-existing decision stage or from the insertion of such a stage on only some proportion of trials.

From an analysis of the standard deviation of the responses and how they change with SOA ( Figure 4A), we can infer the following facts: (1) At small SOA, the response time to the second task (dashed line) is more variable than the response task to the first task (dotted line). (2) The difference in response times (RT2-RT1) is less variable than the response time to each individual task, indicating that variability of the second response does not result just from the concatenation of two equally variable processes. This is consistent with the strong correlation observed between both responses at short SOAs ( Figure 4B). The weak variability of RT2-RT1 is inconsistent with a model of sequential task concatenation, thus constituting a third departure from the passive bottleneck model. (3) Even for large SOAs (500 ms), the standard deviation of the second task remains larger than that of the first task, and the standard deviation of the difference of response times remains less than each of the individual processes. Furthermore, there is still a significant correlation between RT1 and RT2. Altogether, this indicates that even at this SOA, both responses manifest a strong interaction. This provides yet another indication that there is a significant interaction between both responses even at SOA of 1,000 ms, indicating that the regime of interference extends well beyond the boundaries of what had been found in interference experiments with fixed task order. Note that this correlation cannot be accounted by a slow attentional drift, since the correlation between consecutive trials is C = 0.1, significantly smaller than the correlation between RT1 and RT2 at an SOA of 1,000 ms.

**Figure 4:**
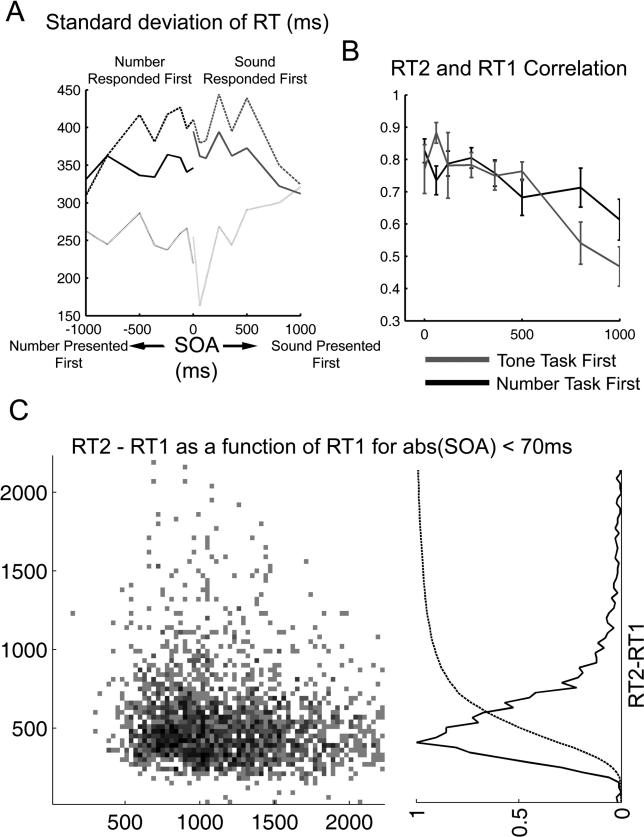
Standard Deviation and Correlation of the Responses to Both Tasks (A) Standard deviation of response times to the first and second task, and of the difference in response times. For short SOAs, the response time to the second task is more variable, consistent with a model of accumulation of variance in successive stages. The variance of RT2-RT1 is considerable smaller than the variance of RT1 and RT2 at long SOA indicating a strong correlation between the two responses. (B) An explicit measure of the correlation between RT1 and RT2 shows a monotonic decrease with SOA, but even for a SOA of 1,000 ms, they are strongly correlated. This correlation cannot be accounted for by momentary drifts in participants' attention since the correlation between response times of consecutive trials is 0.08 ± 0.06 ms. (C) The left panel shows a histogram of occurrences of RT2-RT1 as a function of RT1. All the data corresponding to SOA values of −60, 0, or 60 ms (simultaneous or quasi-simultaneous presentation) were grouped and binned in windows of 30 × 30 ms. For reference, a line indicates the value of RT2-RT1 = 150 ms. Almost no responses fall below this line. On the right is shown the histogram and the cumulative distribution of RT2-RT1 (collapsed across all values of RT1).

A simple interpretation for the low variability of RT2-RT1 could be the existence of a fraction of “grouped” trials. Grouping refers to several possible strategies that share the core idea that the two responses are coupled in some way. For example, the dual task might be treated as a single compound stimulus S1 + S2 with a corresponding compound response R1 + R2 [ 31]. Alternatively, the first response could be deferred until the second response has been selected, so that the two responses can be emitted in rapid succession [ 32]. In both alternatives, all the processing stages of both tasks are performed before the execution of the first response, and hence both responses should be very tightly correlated. There are, however, a number of arguments that suggest that a substantial amount of grouping in this data is highly unlikely: (1) Experiments in which responses were voluntarily grouped have observed a very short delay between responses, typically less than 100 ms [ 7, 33]. By contrast, in our experiment, less than 0.2% of the trials show a difference below 100 ms. ( Figure 4C); (2) in grouped trials, the response to the first task is not executed until the second task is performed. This implies that RT1 increases monotonically with SOA. In our data, we do not observe this behavior. While it can still be argued that the amount of grouping is in itself a function of SOA (for example, there may be more grouping when both tasks are presented closely), the particular non-linear dependence that we observe (which ramps at an SOA of 300 ms and then plateaus) makes this possibility extremely unlikely; and (3) If mean RT1 is slow at short SOAs due to a proportion of grouped trials, a correlation would be expected between RT2-RT1 and RT1. This is expected since grouped trials would correspond simultaneously to the slower RT1 and to the faster RT2-RT1. Instead, in our data, RT2-RT1 is completely independent of RT1 ( Figure 4C; a linear regression between RT2-RT1 and RT1 gives a slope of −0.068±0.09 and *R ^2^* = 0.02 ± 0.006. Both measures were calculated for each individual participant and then averaged).

## Discussion

To clarify the relationship between task chronometry and task choice, we studied a dual-task paradigm in which participants could freely select the task response order while we manipulated the difficulty of one of the two tasks. Although we still observed the main features of the PRP paradigm, suggesting a serial execution of the central stages of the two tasks, our experiment also led to three observations that cannot be accounted for by a passive central bottleneck model: the slowing of RT1, the absorption of central costs of the second task, and the high correlation of responses to the two tasks. All of these effects occur specifically at short SOAs. We argue that they constitute the signature of active processes of task setting (planning of the appropriate sequence of actions) and task disengaging (suppression of the plan set for the first task in order to proceed with the next one). We now show how our complex data can be explained by an extended, hierarchical model of cognitive architecture that combines dynamically these different manifestations of central processing. Our model relates basic structural limitations (i.e., the fact that certain processes cannot be executed simultaneously) with central executive variables, constituting a first step towards generating realistic and self-contained models of human behavior acting in a dynamic environment in which task choice, initiation, and execution are interlinked.

### Chronometry of Task Choice

A series of studies have attempted to establish a formal relation between the temporal organization of the different subprocesses that constitute a task and their serial or parallel nature [ 3, 9, 27, 34– 43]. Several theories propose that there is a first, peripheral stage of parallel information processing followed by a second, central stage of serial processing [ 8, 36]. A natural question to ask is whether, when two streams are competing for access to this central system, the outcome of the competition is decided upon stimulus presentation or rather after the perceptual peripheral processing has been completed. Our first finding argues that the outcome of this competition is not fully decided at the time of stimulus presentation and that a task for which the first peripheral stage of processing is fast has an advantage. We conclude this by observing that the choice of task response order varies with the notation, but not with the distance manipulation of the number task. This separation is important because it shows that task order is not just affected by the “complexity” or total response time of a given task, but more precisely by the duration of perceptual processing, since we had previously shown that the notation manipulation affects a peripheral perceptual stage and the distance manipulation a central, decisional stage.

Although this result argues for a selection of access to central space after a stage of peripheral perceptual processing, a purely passive, first-come, first-served model of access to the central stage cannot suffice for several reasons: (1) The shift of the 50%SOA between both notations (37.8 ms) is considerably smaller than the response time change resulting from this notation manipulation (86 ms); (2) the size of the notation effect and the shift in 50%SOA show a weak correlation ( *r* = 0.55) across participants, indicating that on a participant-by-participant basis, differences in performance across notation cannot fully account for the shift in 50%SOA; (3) the dispersion of choice probability over SOAs is approximately 300 ms, even within individual participants. Previously we have shown that the variability in the duration of perceptual processes is less than a few tens of milliseconds and thus fluctuations in choice cannot be explained solely on the basis of delays in perceptual processing lines; (4) this dispersion is such that, even for moderately long SOAs (up to 300 ms), there is a significant proportion of trials in which participants execute the tasks in the order opposite to the order of stimulus presentation; and (5) in other experimental designs, when instructed to respond always in the same order, participants can set a fixed order of response for simultaneous or delayed presentations at no cost (in RT), indicating that task selection can be determined fully by top-down control.

Altogether, these arguments point to an interaction between bottom-up factors and a top-down decision process in determining which task is performed first. In the model developed further below, we will assume that task setting is the result of a slow, stochastic, and possibly biased decision process that weights the evidence in favor of the presence of one or the other stimulus before assigning central resources to one or the other task.

### A Minimal Model for Response Times in Dual-Task Paradigms: Incorporating Task-Switching Costs to Sequential Processing Models

In the present experiment, participants were explicitly asked to perform a dual task. Yet, they were implicitly performing another decision necessary to achieve their goal, namely, in which order to perform the two tasks? We hypothesize that in this particular experimental situation, we should observe a trace of a hierarchy of nested processes [ 4, 44– 47]; first setting the system for a particular task execution order, and then, within each task, executing the series of subprocesses that compose it. We now show how such a structure can account for the three critical departures that have been highlighted in the results section. The basics of the model are described in Figure 5. The passive bottleneck model [ 8, 9, 24, 27, 37, 48] constitutes the starting point. The first modification, the insertion of an initial task-setting process, is required to account for the dependency of RT1 on SOA. In a previous experiment [ 27], we showed that to provide an accurate description of response times for both tasks, a fixed time thought to be related to executive processing and engaging of the system needed to be added. Here we found that in a situation in which task order is unpredictable, this time increases as SOA gets shorter, suggesting that task setting is more difficult when task choice poses a conflict. This is consistent with previous findings of the role of preparation on overlapping-task performance, which have suggested that when task order is known, sequential performance of overlapping tasks is scheduled in advance [ 49].

**Figure 5:**
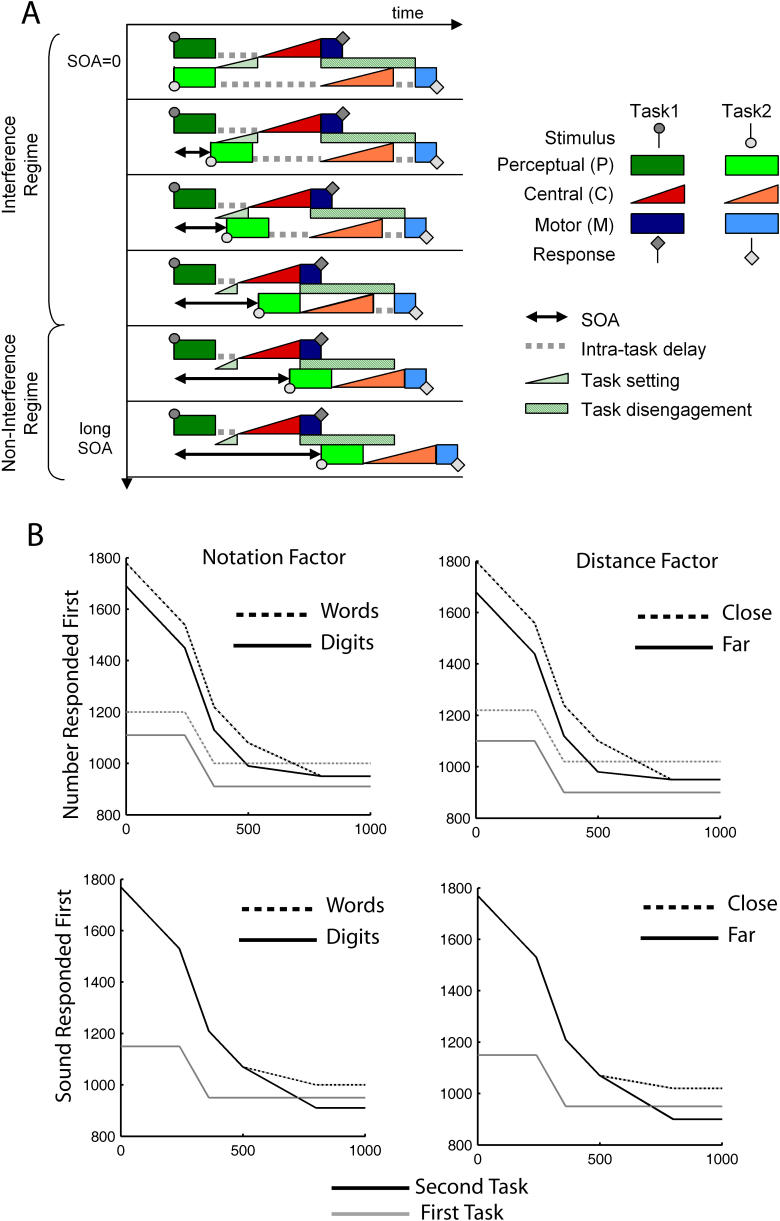
A Minimal Model for Dual-Task Response Times: Incorporating Task Switching Costs into Sequential Processing Models (A) Minimal model capable of accounting for the critical observations in our interference paradigm (see Results and Discussion for a more detailed description). Each task consists of three basic processing stages: perceptual (P), central (C), and motor (M) processes. It is assumed that only the central process establishes a bottleneck whereas other stages can be carried out in parallel with stages of another concurrent task [ 8, 24]. Central processes, which are assumed to rely on stochastic evidence accumulation mechanisms and therefore make a major contribution to response-time variability [ 27], are depicted with triangles. Non-decision processes, which have a relatively fixed duration, are depicted with boxes. The model supposes that a first central decision is required to select which task to perform first. We refer to this stage as *task setting*. We assume that its duration is longer at short SOAs, when both stimuli are in competition, than at long SOAs, when a single stimulus is presented. A second postulate is that there is a temporary inhibition of the response to the second task, implying that it cannot be executed until the first task has been disengaged, as observed in task-switching paradigms [ 14]. We refer to this stage as *task disengagement*. Note how, during the interference regime, all three central decisional processes (triangles) follow each other in time, indicating saturation of the central system, which causes the observed response delays (dashed lines). Those delays essentially vanish as SOA increases. (B) Pattern of results predicted by the model (same format as in Figure 2B). All the key observations can be fitted with a single set of parameters. The stage durations, in milliseconds, are: P(Number) = 350, P(Tone) = 320, C(Number) = 530, C(Tone) = 580, M(Number) = 50, M(Tone) = 30, Task Disengagement = 600, and Max Task Setting = 200. The fit yields a mean square error (averaged across all conditions) of 42 ms.

A second addition to the passive bottleneck model of the PRP, the insertion of a task-disengagement process, is necessary to explain the other two major experimental departures from the sequential model. Although we had found previously that the distance manipulation affected a central stage of the number task [ 27], the absorption of the distance effect in the present data implies that this stage cannot be the only limiting stage. Based on the previous proposal that task inertia is a critical factor in task-switching paradigms [ 14, 18, 19], we have included another limiting stage of disengagement of the concurrent task. Although central processing of task 2 can be executed immediately after central processing of task 1 has been completed, the outcome of task 2 cannot be executed until the system has disengaged from the previous response-setting mode. Such an organization is analogous to previous models of cognitive architecture that postulate two bottlenecks, one due to response selection and the other to response initiation and modulated by the type of effector used to respond [ 33, 50].

Once task-setting and disengagement costs are added, our revised bottleneck model can account for the remaining findings that were found inconsistent with the passive bottleneck model. Concerning first the absorption of the distance effect, augmenting the duration of the central stage of task 2 does not affect the response because, for short SOA, the limiting stage is task disengagement. Second, the strong correlation between RT1 and RT2 for short SOA and the weak dispersion of RT2-RT1 can be explained since both responses are locked in time by a single disengaging process which may have a relatively invariant duration.

While other alternatives may provide an explanation for some of these findings, they do not seem to be able to explain simultaneously all of our observations. For instance, the central capacity sharing model [ 51– 53] suggests that central processing of multiple tasks can be carried out simultaneously, but with a limited total capacity. This model correctly predicts an increase in RT1 for short SOAs. However, many of our other observations are incompatible with central capacity sharing. First, the model predicts an interaction between SOA and a central manipulation of task 1, since the additional central processing must be carried out under reduced capacity at short SOAs but not at long SOAs. However, in our task, when the number task is responded to first and the central factor of numerical distance is manipulated, we do not observe such an interaction. Likewise, the model is unable to explain the disappearance of the distance effect for RT2 at short SOAs when the tone task is responded to first. Finally when the number task is responded to second, changing the notation (a pre-bottleneck stage) postpones central processing. Thus, according to the central capacity sharing model, the point at which the first task must relinquish some capacity is postponed and thus it should be speeded up. In our task, responses to the tone task when presented first are independent of the notation of the number task.

Overall, despite the simplicity of our experimental design, the resulting data are difficult to capture by a very simple model. We do not argue that the model we propose is the only possible one, or even the optimal one. Such a conclusion is clearly beyond reach when the space of possible models incorporates so many dimensions. We have merely built the simplest model we could conceive of that could explain all the data and remain consistent with previous findings. Indeed, we started from a highly predictive model in a more simplified situation, and all the components that we have incorporated appear necessary to explain our observations.

### Which Generalizations May or May Not Be Drawn from the Present Findings?

The present experiment does not constitute an exhaustive analysis of the different dimensions of task choice and processing interference. For example, there is no doubt that the saliency of the stimuli (luminance or volume), the participants' expertise, and other factors might influence task response order. Here we have concentrated on the influence of the duration of different processing stages of one task, and left all other factors unchanged. Similarly, this experiment does not provide a complete factorial exploration of all the possible sources of processing interference. For example, we have explicitly avoided low-level sensory interference by presenting stimuli in two different modalities. On the motor side, although we have used different hands to avoid low-level motor interference, it is very likely that some of our observations are specific to bimanual responses and would not necessarily transfer to multi-modal motor responses, for instance hand–voice or hand–feet combinations [ 54]. Indeed, the disengagement cost is present in bimanual responses but seems reduced or absent in other motor configurations [ 33, 54], in agreement with a hierarchical organization of motor preparation and action [ 55– 57]. It is possible that, in our task, the response is first planned at a spatial representation level, in which the leftward or rightward direction is encoded, and only subsequently assigned to the appropriate effector hand. Evidence supporting this hierarchical organization comes from learning studies that show that a learned sequence transfers across hands but not to different sequences within the same hand [ 58].

The relative contributions to response time of the different components of the model and their dependence on experimental factors might also vary with the specific experimental setup. For example, in fixed-order dual-task experiments, there is no trial-to-trial task-order decision and thus the task-setting cost is independent of SOA. Regarding task disengagement, we think that the time to disengage from one action to another may also critically depend on experience and may become quite short under fixed task-order instructions [ 59]. Note from the model of Figure 5 that if the duration of task disengagement is less than the central component of the second task, then it should have no observable consequences. Essentially, the measure of interference is determined by the maximum of two limiting factors: the bottleneck duration and the disengagement duration.

Finally, in some circumstances, central interference can become negligible [ 60– 62]. While central interference persists in multimodal dual-task experiments [ 54], it can be greatly reduced when the two tasks use distinct and highly automated stimulus-response mapping [ 63, 64]. However, recent results suggest that even under conditions of high ideomotor compatibility, the locus of the central processing bottleneck may be shifted but not completely eliminated [ 65]. This is consistent with the idea that learning may result in a shift of the cortical representation of complex features toward earlier stages in the processing pathway [ 43, 66, 67], which may provide a neural implementation for the process of automatization [ 68, 69]. This implies in particular that the serial or parallel character of certain processing stages may be, at least in part, a matter of experience.

### Possible Cerebral Substrates of the Different Components

A common feature that has emerged from several efforts devoted to understanding the architecture of information processing in the human brain is the existence of two qualitatively different types of neural processes: modular (parallel) and central (serial). In different cognitive theories, the central process has been named the central executive [ 2], the supervisory attentional system [ 4], the anterior attention system [ 3], the capacity limited stage [ 36], the global workspace [ 1, 70], or the dynamic core [ 71]. Its engagement appears to be imperative for numerous cognitive functions such as executive and effortful mental action, working memory, and conscious processing. It is characterized by certain basic dynamic and architectonic features: (1) It collects information from many different modules, (2) it cannot proceed in parallel with other processes of the same type, (3) it is sustained for a few hundred of milliseconds, and (4) it is highly stochastic.

Behavioral experiments which have combined the basic features of different manifestations of central processing such as the PRP (two rapid responses) or the attentional blink (extinction of a second rapidly presented stimulus) have shown that both forms of processing limitations may arise in part from a common bottleneck [ 37, 72, 73]. Indeed, more recent neuroimaging data have argued for a role of parietal and prefrontal areas in central processing both in the attentional blink (AB) [ 36, 74, 75] and in the PRP [ 37, 76, 77]. More generally, several different lines of evidence have identified an extended network which includes parietal and lateral frontal cortex as a neural candidate of central processes [ 4, 47, 75, 78], involved in stimulus-response mapping [ 79], necessary in effortful but not in automatic tasks [ 80] and ubiquitously present in a large variety of goal-directed tasks [ 81].

Our model suggests that during dual-task processing, three central stochastic decisional processes follow each other without any temporal gap: task choice, selection of first response, and selection of the second response (depicted by triangles in Figure 5). Each decision-making process has been modeled as a noisy integrator [ 82– 86] that accumulates evidence provided by the sensory system in a highly stochastic manner and is responsible for most of the trial-to-trial variance in response times [ 27, 87]. Neural integration has been observed in multiple cortical areas including posterior parietal, dorsolateral prefrontal, and frontal eye fields [ 87, 88], thus overlapping largely with the nodes previously associated with central processing by dual-task experiments.

Although much evidence thus points to a distributed parieto-frontal network, parsing out the different contributions to central processing within this large network remains an important challenge. Our model provides predictions and experimental directions to achieve this goal. According to our model, different contributions to central processing may be parsed according to their susceptibility to experimental manipulations and to intrinsic trial-to-trial variability within a fixed experimental condition. A first prediction is that modulations of the engaging and disengaging stages should result in a differential activation in the dual-task condition relative to the sum of activation observed in single tasks. In contrast, the passive bottleneck model predicts no change in the total activation of the central component of each task (just a dynamic reorganization) and thus predicts that this contrast should not evoke a difference in the fMRI signal. Moreover, the duration of the task decision process should depend on task-order knowledge and should show a strong covariation with RT1 but not with the interval between both responses (RT2-RT1). Compatible with part of those predictions, a recent fMRI study that manipulated task order in a PRP experiment showed that cortical areas along the posterior part of the left inferior frontal sulcus were more strongly activated in different-order than in same-order trials [ 89].

Our proposed cognitive architecture emerges from a synthesis of three major sources: passive bottleneck models [ 7, 8]; models of task switching costs [ 14, 18, 20] and of attentional inertia [ 22, 23, 90]; and hierarchical models of cognitive architecture [ 4, 44, 46, 47, 91, 92]. By specifying the organization, response delay, and response variability of those mental processes, down to a level of detail where the full response time distribution can be accounted for [ 27], our research paves the way for an identification of their cerebral correlates.

## Materials and Methods

### Participants.

A total of 16 participants, all right handed, were involved in this study (eight males, age 21 ± 3 y). Participants were all native French speakers and were remunerated for their participation.

### Procedure.

Participants were asked to perform two tasks, with the clear instruction that they had to respond accurately and as fast as possible to each one as it arrived. The delay in the onset of the two tasks changed randomly from trial to trial from 0 ms (simultaneous presentation) to 1,000 ms. In the number comparison task, a number was flashed in the center of the screen for 150 ms, and participants had to respond as to whether the number was larger or smaller than 45. The presented numbers ranged between 21 and 69, excluding 45. In different trials, the number was presented in Arabic digits or in words. Participants responded to the number task with a single key press using the right hand; the middle finger to indicate that the number was larger than 45 and the index finger to indicate that it was smaller. In the tone task, participants were asked to discriminate between a low (440 Hz) and a high (880 Hz) tone, both lasting 150 ms. Participants responded with a single key press with the left hand; the middle finger to indicate the low tone and the index finger to indicate the high tone. The numerical distance between the target and 45, and the delay between the presentation of the two stimuli, varied randomly, and trials were presented with an inter trial interval (ITI) that jittered (i.e., varied randomly) between 3,100 and 3,300 ms.

In each block, there were 33 different trial types, including 15 different SOA values (0, ±60, ±120, ±240, ±360, ±500, ±800, and ±1,000 ms) for each notation (30 total), the number task presented alone in both notations, and the tone task presented alone. To present the same number of trials for the number task and tone task in isolation (regardless of notation), two trials of the tone task were presented in each cycle. Thus, each cycle consisted of 34 trials: 30 double-task, two number task alone, and two tone task alone. Participants performed a total of 15 cycles (a total of 510 trials) divided into five blocks with pauses in between blocks. Before starting data collection, participants performed a training block of three cycles (102 trials).

### Stimuli.

Stimuli were shown in a black and white display in a 17-in monitor with a refresh rate of 60 Hz. Participants sat 1 m from the screen. Stimuli were always presented in the fovea, and their size was 1° for the Arabic digits and 2.5° for the words. Auditory stimuli were pure tones of 150 ms duration and 440 or 880 Hz frequency. Auditory stimulation was provided through headphones.

### Data analysis.

All the analyses described here were done only on correct responses. In dual-task trials, a trial was considered correct if participants responded correctly to both tasks, regardless of task order. Since there were two tasks and each task had two possible responses, chance level for this experiment is at 25%. Errors (15%) include errors either to the first or second task, and trials where participants failed to respond to either of the tasks, or both. Trials in which the response times to either task were longer than 2,000 ms (<4% of the trials) were excluded. All the statistics were done using the R software package, and in all ANOVAs, participants were treated as a random factor. 50%SOA ( Figure 1) was calculated on a participant by participant basis, by linearly extrapolating the distribution to find the 50% crossing. Correlations between RT1 and RT2 ( Figure 4) are measured as the zero lag covariance:



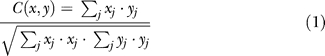



where *j* corresponds to the different observations of a single participant. A measure of correlation was calculated for each individual participant and then averaged across participants.

The model used to fit the data had ten parameters, eight of which were fit in an exhaustive search to generate the distributions that generated mean RT1 and RT2 that departed less (using the minimal squares criterion) from the observed distribution. Mean RTs were calculated according to the formulas:



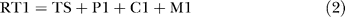





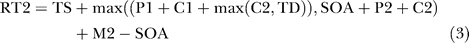



and



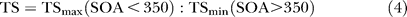



where TS is the duration of the *task setting stage,* which ramps from TS _max_ to TS _min_ at 350 ms. TS _min_ was set to 100 ms. The precise decay form of this function did not result in significant changes in the goodness of the fit. TD is the duration of the *task disengagement stage*. P1, C1, M1, P2, C2, and M2 are the durations of the perceptual, central, and motor components, respectively, of tasks 1 and 2. Same parameters were used to fit both task orders, with fixed values of P, C, and M for the tone and number tasks. All distributions resulting from task order, notation, and distance (a total of eight distributions) were fit simultaneously with fixed parameters. The changes in processing components resulting from the different manipulations were not fit but rather obtained from the effects of each manipulation on response time: For the notation manipulation P(number) was increased by 86 ms and for the distance manipulation C(number) was increased by 112 ms. Thus, a total of ten parameters (eight were fit) were used to fit eight experimental distributions. The mean standard error for the best fit, averaged across all distributions, is 42 ms.

## Abbreviations

ANOVA: analysis of variance
CI: confidence interval
PRP: psychological refractory period
RT: response time
RT1: response time to the first task
RT2: response time to the second task
RT2-RT1: response time to the second task minus the response time to the first task
SOA: stimulus onset asynchrony
